# Saphenovenous Graft Aneurysm: A Rare Complication of CABG

**DOI:** 10.1155/2017/8101489

**Published:** 2017-05-18

**Authors:** James Thomas Connell

**Affiliations:** Royal Adelaide Hospital, North Terrace, Adelaide, SA 5000, Australia

## Abstract

Saphenovenous graft aneurysm is a rare complication of coronary artery bypass grafts that is likely underdiagnosed. It is typically asymptomatic, slow growing, and often diagnosed incidentally on angiography or following catastrophic rupture. There is no consensus on best management but PCI and surgery appear to have more favourable mortality outcomes relative to conservative management. We present the case of a 48-year-old male with a cardiovascular risk profile hallmarked by diabetes mellitus, end stage renal failure, recalcitrant hyperlipidaemia, and IHD previously treated with CABG. 11 years following his CABG, he was retrieved from remote Australia to a tertiary cardiology centre with stabbing chest pain. Serial cardiac enzymes were negative. Echocardiogram identified a mass compressing the right ventricular wall. Noncontrast coronary angiogram ultimately identified a large aneurysm at the proximal end of SVG to PDA. He was managed with aggressive risk factor modification prior to planned surgical intervention once medically optimized. His case supports the role of aggressive medical management combined with surgical intervention.

## 1. Case Report

*Clinical Presentation*. We present the case of a 48-year-old male from remote Australia. He reported a history of subacute onset, sharp, central chest pain, stabbing in nature. It was different in character to his previous angina and persisted for hours. He described no associated dyspnoea, palpitations, or diaphoresis.

His cardiovascular profile was hallmarked by insulin dependent diabetes mellitus, hypertension, end stage renal failure secondary to diabetic nephropathy, recalcitrant hyperlipidaemia, previous cigarette smoking and alcohol intake, and previous coronary artery bypass in 2003. At that time he underwent left internal mammary artery (LIMA) graft to his left anterior descending artery (LAD) and saphenous vein graft (SVG) to his posterior descending artery (PDA). The patient also had a strong family history of ischaemic heart disease, hyperlipidaemia, type 2 diabetes mellitus, and renal failure.

**Figure 1:**
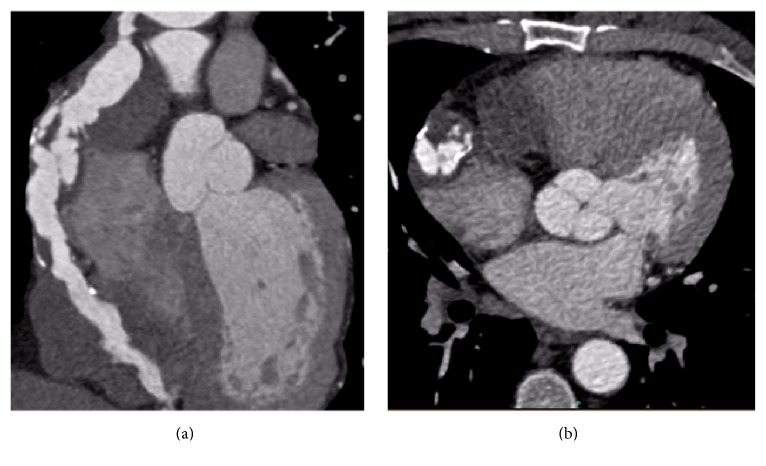
Coronal and axial slices from CT coronary angiogram with the calcified aneurysmal SVG to PDA graft.

**Figure 2:**
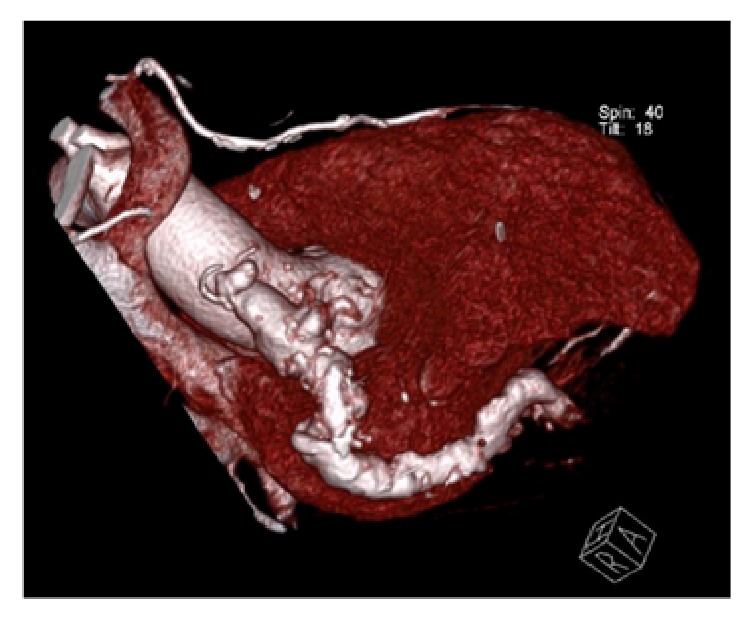
Right anterolateral view of a 3D reconstruction highlighting the aneurismal SVG graft.

*Investigations*. Our patient had negative serial cardiac enzymes. ECG showed subtle anterolateral T wave inversion. He underwent an echocardiogram with an unanticipated and initially difficult to interpret finding. There was a 4.5 × 5 cm mass intimate and compressing the right ventricular wall. In the context of his ESRF, a noncontrast CT was undertaken to further quantify the lesion. This was suggestive of a large vascular structure with calcified walls, in keeping with an aneurysm. The origin of the vascular structure was indeterminate and the patient ultimately underwent a coronary angiogram (Figures 1(a) and 1(b)). A mid segment left circumflex (LCX) lesion was successfully stented with a drug eluting stent. LIMA to LAD was found to be patent. Angiography of his SVG to PDA identified a unique finding. There was a diffusely ectatic large aneurysm at the proximal end of his SVG to PDA (Figures 1(a), 1(b), and 2).

*Management/Follow-Up*. His case was discussed at a combined cardiology and cardiothoracic multidisciplinary meeting. The consensus was to pursue conservative management in the first instance and consider surgical intervention at a later date when medical management had been optimized. Aggressive risk factor modification was undertaken, particularly with regard to controlling his recalcitrant hyperlipidaemia. He remained under the surveillance of the cardiology department. After 18 months he returned for elective surgical intervention by ligation with bypass. His procedure was completed without complication and he recovered in cardiothoracic intensive care unit. He was discharged after 5 days and remains well at 6 months.

## 2. Discussion

In 2012, Ramirez et al. undertook a literature review analyzing all published cases of saphenous vein graft aneurysms since the first documented case in 1975. There were 168 publications comprising small case series and single case studies. There were 209 cases of SVG aneurysms included in the literature review [1]. One single centre retrospective study estimated incidence to be 0.07% based on 4 SVG aneurysms in a cohort of over 5500 cases of CABG that underwent surveillance over decades [2]. Whilst reported cases are extremely rare, their incidence is likely understated due to

- typically asymptomatic clinical course,
- incidental findings on imaging or angiogram,
- no current screening guidelines,
- the potential for them to be missed on angiography if the lesion contains significant thrombus burden,
- the fact that first presentation may be sudden death from rupture.

Ramirez et al. found that they not only are rare but also are typically a late complication. 90% of cases are found greater than 5 years after bypass surgery with the mean time of diagnosis being 13.2 years after grafting [1].

There were 9 publications that reported surveillance on aneurysm size. All 9 had a common finding of aneurismal growth over time [1]. The relationship between aneurysm size and rates of complication demonstrated a positive correlation between SVG aneurysm size and incidence of adverse outcomes. As aneurysm diameter approached 10 mm, adverse events increased proportionally. All-cause mortality within 30 days of diagnosis was reported at 13.9% [1].

The most commonly observed treatment option has been surgery; however PCI has become increasingly popular over the past decade. Surgery has been advocated whenever compressive complications are present, when there is compromised myocardium that would benefit from bypass, and when the candidate is fit enough for an operation [3, 4]. The surgical techniques used have been aneurismal ligation or aneurismal resection plus/minus bypass revascularization [1, 3].

Conservative management has been reported in 20% of patients. The approach has been secondary prevention, risk factor modification, and surveillance [5]. Mortality rates were higher in the conservative management specific cohort.

PCI has been a popular modality with more favourable mortality rates. It has been the least trialed treatment pathway with 32 published cases. Coiled embolization, septal occlusion, and stenting have all been employed [1, 3, 6].

Ramirez et al. concluded that surgery and PCI were viable treatment options for SVG aneurysms. The high rates of complications in even small aneurysms meant surveillance or conservative management was less appropriate [2]. They conceded that the number of small, stable aneurysms may be underdiagnosed and therefore complications and mortality incidence may be overrepresented in the conservative cohort [1, 7].

Our case provides an example of a unique and underdiagnosed complication of CABG. The optimal management of SVG graft aneurysm has not yet been established, but there appears to be benefit from a combination medical and surgical intervention. Our patient was a high-risk surgical candidate who was relatively asymptomatic. By careful risk stratification we determined that aggressive medical management of his comorbidities was appropriate prior to ultimately proceeding to definitive surgical intervention.

## References

[1] Ramirez F. D., Hibbert B., Simard T. (2012). Natural history and management of aortocoronary saphenous vein graft aneurysms: a systematic review of published cases. *Circulation*.

[2] Dieter R. S., Patel A. K., Yandow D. (2003). Conservative vs. invasive treatment of aortocoronary saphenous vein graft aneurysms: treatment algorithm based upon a large series. *Cardiovascular Surgery*.

[3] Sareyyupoglu B., Schaff H. V., Ucar I., Sundt T. M., Dearani J. A., Park S. J. (2009). Surgical treatment of saphenous vein graft aneurysms after coronary artery revascularization. *Annals of Thoracic Surgery*.

[4] Memon A. Q., Huang R. I., Marcus F., Xavier L., Alpert J. (2003). Saphenous vein graft aneurysm: case report and review. *Cardiology in Review*.

[5] Wojciechowski A., Skowronek P. (2016). Aortocoronary saphenous-vein-graft aneurysms. *New England Journal of Medicine*.

[6] Nölke L., McGovern E., Wood A. E. (2004). Saphenous vein graft aneurysms; the true, false and ugly!. *Interactive Cardiovascular and Thoracic Surgery*.

[7] Jorgensen JP., Helmy T., Yang EP. (2014). Saphenous venous graft aneurysms. *Medscape eMedicine*.

